# Inflammation, Immunonutritive, and Cardiovascular Risk Biomarkers in Men With Alcohol Use Disorder

**DOI:** 10.7759/cureus.59522

**Published:** 2024-05-02

**Authors:** Nilifer Gürbüzer, Elif Özcan Tozoğlu

**Affiliations:** 1 Department of Psychiatry, Erzurum City Hospital, Erzurum, TUR

**Keywords:** c-reactive protein-albumin-lymphocyte index, plasma atherogenicity index, systemic immune response index, systemic immune inflammation index, alcohol use disorder

## Abstract

Objective: Alcohol Use Disorder (AUD) is a significant public health issue associated with serious health risks. This study aims to reveal the relationship between AUD and inflammatory, immunonutritive, and cardiovascular risk markers by evaluating hemogram and biochemistry parameters together in AUD.

Method: The data of 54 male patients with AUD and 45 male controls were included in the study. Sociodemographic-clinical data of the participants and Alcohol Use Disorders Identification Test (AUDIT) results were obtained from medical records. Systemic immune inflammation index (SII) was obtained with the platelet x neutrophil/lymphocyte formula; systemic immune response index (SIRI) was obtained with the monocyte x neutrophil/lymphocyte formula, plasma atherogenicity index (AIP) was obtained with the ratio of triglyceride to High-density Lipoprotein (HDL) cholesterol. C-Reactive Protein (CRP) albumin-lymphocyte (CALLY) index was obtained with the albumin x lymphocyte/CRP x 10^4^ formula.

Results: Aspartate aminotransferase (AST), Gamma Glutamyl Transferase (GGT) activities, neutrophil, CRP, ferritin, SII, and SIRI levels were significantly higher in those with AUD compared to controls. Laboratory results of those with AUD were consistent with atherogenic dyslipidemia; higher triglyceride and total cholesterol levels and AIP values were found compared to controls. The amount of alcohol consumed was a predictor for high SII, SIRI, and AIP levels. The CALLY index, which evaluates immune function, inflammation, and nutritional status together, was significantly lower in patients compared to controls. The amount of alcohol use and the total AUDIT score were predictors for a low CALLY index.

Conclusion: The results of this study support that AUD is a chronic inflammatory psychiatric disorder. We suggest that new inflammatory, immunonutritive, and cardiovascular biomarkers SII, SIRI, AIP, and CALLY index could be promising clinical tools to evaluate the severity, potential complications, and treatment response of AUD.

## Introduction

Alcohol Use Disorder (AUD) is listed under the heading of substance-related disorders and addiction disorders in the 5th Edition of the Diagnostic and Statistical Manual of Mental Disorders (DSM-5). AUD is defined as problematic alcohol use that causes clinically significant distress or decline in functionality in a person’s life [1]. This disorder is a significant public health problem associated with health risks, including negative medical, psychological, and functional impairment and premature death [2,3]. The negative effects of alcohol on a person’s health depend on the amount, type, and way of consumption of alcohol [4-6]. Although recent studies have focused on the beneficial effects of mild to moderate alcohol consumption [5], long-term high alcohol consumption continues to be among the leading causes of deaths due to liver diseases and cardiovascular diseases (CVD) [4,5,7].

It has been suggested that alcohol can demonstrate its harmful effects on the cardiovascular system through three mechanisms: risk factors (for example, lipid profiles), hemostatic factors (for example, platelet reactivity), and inflammation. Long-term high-level alcohol consumption increases blood lipids and causes hypertriglyceridemia. High triglyceride levels are associated with atherosclerosis and consequently increased CVD risk in the literature [4,6]. A recent study has shown that long-term excessive alcohol consumption causes inflammation and atherogenic dyslipidemia [4]. Studies show that atherosclerosis is not only a lipid accumulation disease but also involves a chronic inflammatory process [8]. It has been suggested that inflammatory markers such as C-reactive protein (CRP) could be a helpful method for the overall assessment of CVD risk, and high CRP levels have been shown to be a strong and independent predictor of CVD risk in apparently healthy individuals [9].

Inflammation is a physiological process that helps repair tissue damage and eliminate infections. However, abnormal responses or chronic inflammation can be pathological [7]. Neutrophils are a traditional hematological index reflecting the inflammatory state of the immune system. Monocytes contribute to immune defense and the damage-repair process, lymphocytes contribute to the regulation of the immune system [10]. The majority of psychopathologies have been associated with inflammation and/or changes in the immune system [11]. Studies associating inflammation and atherogenic dyslipidemia with AUD are available in the literature [2,4,12-15]. Blood biomarker studies are the most commonly used study method in general practice; however, the routine collection of many of these biomarkers is difficult or expensive to measure, which can limit practical application in the clinic. Affordable ones, such as platelet count, white blood cell count, subtypes and their ratios (systemic immune inflammation index-SII, systemic immune response index-SIRI), CRP, serum lipid profile, their ratios (plasma atherogenicity index-AIP) are determinants of inflammation and cardiovascular risk in psychopathologies including AUD [2,4,13,15,16]. The CRP-albumin-lymphocyte (CALLY) index was first defined by Iida and colleagues in cancer patients to reflect the level of inflammation, nutritional status, and immune function, and it has been reported to be a prognostic biomarker [17]. The use of such biomarkers in AUD will contribute to our knowledge about the effects of the amount and duration of alcohol consumed on various organs, tissues, and physiological processes, in addition to verifying the information reported by the individual.

Clinical diagnosis in AUD largely relies on addiction scales. In the diagnosis of patients, organ damage, and treatment follow-up, direct and indirect biomarkers are included; ethanol and its metabolites, liver function tests (alanine aminotransferase (ALT), aspartate aminotransferase (AST), gamma glutamyl transferase (GGT)), lipid profile, their ratios, albumin levels, markers such as transferrin with carbohydrate deficiency [3,18]. Factors such as age, gender, time elapsed since the last alcohol consumption, different drinking styles, use of illegal substances, liver diseases due to other etiologies besides alcohol, CVD, obesity, and anemia can affect the specificity of these biomarkers [3,18]. To improve and enhance the therapeutic process in AUD, there is a need to identify more specific and sensitive markers, especially biomarkers that are easily measurable, cost-effective, highly reproducible, reflect the clinical progression of the disease, and are easy for the clinician to interpret. Considering the low cost, easy accessibility, and clinical usability of the inflammation and nutrition-based SII, SIRI, AIP, and CALLY index, these markers could be good biomarkers in tracking the severity, complications, and treatment response of patients with AUD. When we reviewed the literature, we saw that there are relatively few studies associated with SII and SIRI in AUD and no studies investigating the clinical importance of the CALLY index. The aim of our study is to reveal the relationship between inflammatory, immunonutritive, and cardiovascular risk markers and AUD by evaluating the biochemistry and peripheral hemogram parameters, which are a new, easy, and cheap option in AUD; neutrophil, lymphocyte, monocyte, and CRP levels, liver function tests, lipid profile, SII, SIRI, AIP, and CALLY index together. It is also to evaluate the effect of the clinical characteristics of individuals with AUD on these markers.

In light of all this information, the research questions of this study are as follows: (a) Is there a difference between the biochemistry and peripheral hemogram parameters of individuals with AUD and controls; neutrophil, lymphocyte, monocyte, and CRP levels, liver function tests, lipid profile, SII, SIRI, AIP, and CALLY index? (b) Is there a relationship between age, body mass index (BMI), the amount of alcohol consumed (standard drinks/per week), duration (moon) and other clinical features with inflammatory, immunonutritive and cardiovascular risk markers in AUD? (c) If there is, what is the direction of this relationship and what benefits can it provide in clinical practice?

## Materials and methods

Research design

This retrospective and cross-sectional case-control study included adult male patients who applied to the Erzurum City Hospital Adult Detoxification Center for treatment between January 1, 2022, and December 31, 2023, were diagnosed with AUD according to DSM-5 diagnostic criteria, and met the inclusion-exclusion criteria. The control group was composed of individuals who applied to the psychiatry outpatient clinic for various reasons (such as consultation, a health report stating the situation) and who did not have any physical and mental disability in their medical records, did not have a major psychiatric pathology, and did not use psychotropic drugs.

The research protocol of the study was approved by the Scientific Research Ethics Committee of the Health Sciences University Erzurum Faculty of Medicine (Erzurum, Turkey) with the decision numbered BAEK 2024/03-76 and was conducted in accordance with the Helsinki Declaration.

Research sample

Data from 89 patients were obtained from medical records within the specified date range for the research. The data of 13 patients who used illegal substances along with alcohol and 16 patients with acute/chronic diseases (such as diabetes, hypertension, and acute/chronic inflammatory diseases) were not included in the study. Of the remaining 60 patients, six were female. As the number of female patients was low and it was thought that it would negatively affect the analysis results, the data of female patients were also excluded from the study. The data from a total of 54 patients met the research criteria. In the end, 54 male patients and 45 male control data that met the inclusion and exclusion criteria were included in the study.

The inclusion criterion for patients was to be diagnosed with alcohol use disorder according to DSM-5 diagnostic criteria and not to have any additional psychopathology from a psychiatric perspective. The inclusion criteria for both patients and controls were: being between the ages of 18 and 65, not being obese (BMI < 30 kg/m^2^), not having any additional psychopathology from a psychiatric perspective, not having a mental disability, not having an acute and/or chronic medical and/or autoimmune/inflammatory disease, not having a diagnosis of infectious diseases at the time of sample collection and three months prior, not using antibiotics, probiotics, corticosteroids or other immunomodulators. Since smoking is common in alcohol use disorder, smoking was not accepted as an exclusion criterion for both groups. The clinical and sociodemographic data of the participants were obtained from outpatient clinic records.

Data collection tools

Sociodemographic-clinical data form: All participants’ medical records were reviewed. Features such as age, education level, marital status, employment status, smoking, height, weight, and BMI were obtained from medical records. Participants’ alcohol use durations (moon) and weekly average alcohol consumption amounts (standard drinks/per week) were recorded. A standardized drink, equivalent to 10-14 grams of ethanol, was accepted as a bottle of beer (350 ml), a glass of wine (150 ml) or a shot of tequila, raki, vodka or whiskey (44 ml) [4,19].

Alcohol Use Disorders Identification Test (AUDIT): It was created by the World Health Organization (WHO) to identify people who use alcohol and measure the harmful effects of alcohol use. The final version of the scale was developed by Babor and colleagues [20]. The scale consists of a total of 10 questions: the first three questions are about drinking frequency, questions 4, 5, and 6 are about addiction symptoms and the last four questions are related to harmful alcohol use. The scale is scored between 0-40. The Turkish validity reliability study of the scale was conducted by Saatçioğlu and colleagues [21]. The test-retest reliability of the scale is .90.

Biochemistry and complete blood count measurement and normal reference ranges: all blood analyses were performed at Erzurum City Hospital Biochemistry Central Laboratory using an automatic hematological analyzer (Sysmex XN-1000) and biochemistry (Atellica) analyzer. Neutrophils 1.8-6.98x10^9^/L, lymphocytes 1.21-3.77x10^9^ L, monocytes 0.29-0.95x10^9^/L, platelets 152-383x10^9^/L, ALT 7-40 U/L, AST 13-40 U/L, GGT 0-73 U/L, high-density lipoprotein (HDL) cholesterol 40-89 mg/dL, low-density lipoprotein (LDL) cholesterol 100-129 mg/dL, triglyceride (TG) 0-200 mg/dL, total cholesterol 0-200 mg/dL, albumin 32-48 g/L, ferritin 22-322 ng/mL and CRP 0-5 mg/L. Cholesterol and TG values were converted from mg/dL to mmol/L (for total cholesterol, LDL, and HDL, the mg/dL value was multiplied by the coefficient 0.0259, and for TG, it was multiplied by the coefficient 0.0113) [22]. SII values were obtained from the hemogram results using the platelet x neutrophil/lymphocyte formula and SIRI values were obtained using the monocyte x neutrophil/lymphocyte formula. AIP was obtained by taking the base 10 logarithm of the molar ratio of plasma TG to HDL cholesterol (AIP=log10(TG/HDL)). The CALLY index was obtained with the formula =Albumin serum level (gr/L) x Lymphocyte count (per mm^3^) /CRP serum level (mg/L) x 10^4^.

Statistical analysis

The analyses of the study were performed using the IBM Statistical Package for Social Sciences (SPSS) software, version 22 (IBM Corp., Armonk, NY). Normality analysis was performed, and it was checked whether the skewness and kurtosis values of all variables were between -2 and +2. These values indicate that the assumption of normality is met [23]. Data were presented as mean, standard deviation, minimum, maximum, percentage, and number. In comparisons between two independent groups, the Independent Samples t-test was used when the condition of normal distribution was met, and the Mann-Whitney U test was used when it was not met. Comparisons between categorical variables were made using the Chi-square test. Correlation analysis and linear regression analysis were performed to evaluate the relationship between quantitative variables. If the condition of normal distribution is met in the comparison of two quantitative variables, Pearson correlation is used, if not, Spearman correlation test is used. Receiver operating characteristic curve (ROC) analysis was performed to determine whether continuous variables can be used in diagnosis and to determine cut-off values. The level of statistical significance was taken as p<0.05.

## Results

Data from 54 male patients with AUD and 45 male controls were included in our study. There was a significant difference in terms of education level between the patient and control group (p<0.001). The amount of alcohol consumed by the patients (standard drinks/per week) was 20.35±7.49, the duration of alcohol use (moon) was 153.26±91.04, and the average total AUDIT scores were 27.06±8.37. The comparison of sociodemographic characteristics of the patient and control groups is shown in Table 1.

**Table 1:** Comparison of sociodemographic characteristics of the patient and control groups Note: p<0.05: Statistical significance level in comparison of groups; AUDIT: Alcohol use disorders identification Test; BMI: Body mass index; Mean± SD: Mean± standard deviation; n: Number of participants; t: Independent sample t-test; χ2: Chi‑square; Z: Mann-Whitney U test.

		Patient Group	Control Group	Mean± SD	χ^2^	t,Z	p
		n %	n %				
Age (year)	Patient Group			40.81±10.23		2.534	0.013
	Control Group			35.56±10.35			
BMI (kg/m^2^)	Patient Group			25.16±3.12		-2.147	0.034
	Control Group			26.63±3.71			
Duration of alcohol use (moon)	Patient Group			153.26±91.04			
	Control Group			0±0			
Amount of alcohol use (standard drinks/per week)	Patient Group			20.35±7.49			
	Control Group			0±0			
AUDIT	Patient Group			27.06±8.37			
	Control Group			0±0			
Marital status	Married	28-(51.9)	30-(66.7)		9.360		0.009
	Single	16-(29.6)	15-(33.3)				
	Widowed, divorced, living apart	10-(18.5)	0-(0)				
Educational status	Primary school	12-(22.2)	0-(0)		32.497		<0.001
	High school	36-(66.7)	17-(37.8)				
	University	6-(11.1)	28-(62.2)				
Occupation	Unemployed	14-(25.9)	3-(6.7)		30.406		<0.001
	Private sector employee	28-(51.9)	7-(15.6)				
	Public employee	12-(22.2)	35-(77.8)				
Smoking	There is	50-(92.6)	21-(46.7)		23.310		<0.001
	No	4-(7.4)	24-(53.3)				
Previous history of substance use	There is	14-(25.9)	0-(0)				
	No	40-(74.1)	45-(100)				

The levels of neutrophils (p=0.007), CRP (p=0.001), ferritin (p<0.001), TG (p<0.001), total cholesterol (p=0.003), activities of AST (p<0.001) and GGT (p<0.001), and levels of SII (p=0.001), SIRI (p<0.001) and AIP (p<0.001) in patients were found to be significantly higher compared to the controls. The CALLY index of the patients (p<0.001) was found to be significantly lower compared to the controls (Table 2).

**Table 2:** Comparison of blood parameters between the patient and control groups Note: p<0.05: Statistical significance level in comparison of groups; ALT: Alanine aminotransferase; AST: Aspartate aminotransferase; BMI: Body mass index; CALLY: C-reactive protein-albumin-lymphocyte; CRP: C-Reactive protein; GGT: Gamma glutamyl transferase; HDL: High-density lipoprotein; AIP: Atherogenic index of plasma; LDL: Low-density lipoprotein; Mean± SD: Mean± standard deviation; SII: Systemic immune inflammation Index; SIRI: Systemic inflammatory response index; t: Independent sample t-test; TG: Triglyceride; Z: Mann-Whitney U test.

	Patient Group	Control Group	t,Z	p
	Mean± SD	Mean± SD		
Neutrophil count x 10 ^9^ / ^L^	4.49±1.54	3.79±0.94	2.774	0.007
Lymphocyte count x 10 ^9^ / ^L^	2.51±0.63	2.73±0.55	-1.872	0.064
Monocyte count x 10 ^9^ / ^L^	0.72±0.25	0.64±0.13	-1.287	0.198
Platelet count x 10 ^9^ / ^L^	262.24±53.77	260.20±47.66	0.198	0.844
Ferritin (ng/mL)	191.05±134.89	106.01±65.94	-4.368	<0.001
ALT (U/L)	41.93±27.71	29.89±8.19	-1.362	0.173
AST (U/L)	41.00±35.83	15.49±3.88	-6.610	<0.001
GGT (U/L)	93.26±119.33	26.58±11.90	-4.787	<0.001
TG (mmol/L)	3.37±0.99	1.53±0.78	-5.101	<0.001
HDL Cholesterol (mmol/L)	1.06±0.25	1.08±0.10	-0.637	0.526
LDL Cholesterol (mmol/L)	2.87±0.67	3.02±0.95	-0.858	0.393
Total Cholesterol (mmol/L)	4.96±1.03	4.33±0.98	3.079	0.003
Albumin (g/L)	41.87±2.46	43.73±1.45	-4.476	<0.001
CRP (mg/L)	4.90±5.10	1.97±1.28	-3.397	0.001
SII	496.04±250.67	360.10±86.00	-3.339	0.001
SIRI	1.32±0.64	0.90±0.26	-3.936	<0.001
AIP	0.33±0.24	0.11±0.20	4.945	<0.001
CALLY index	4.69±3.77	7.74±3.66	-4.063	<0.001

A significant and positive correlation was found between the amount of alcohol consumed by patients (standard drinks/per week) and neutrophil, CRP, TG levels, and GGT activities. A significant and negative correlation was detected between the amount of alcohol consumed (standard drinks/per week) and albumin levels. SII and SIRI showed significant and positive relationships with neutrophil, TG levels, and the amount of alcohol consumed. AIP showed a significant and positive relationship with age, neutrophil, CRP level, GGT activity, the amount of alcohol consumed, total AUDIT score, and SIRI values. The CALLY index showed a significant and negative relationship with GGT activity, TG level, the amount of alcohol consumed, total AUDIT score, SII, SIRI, and AIP values (Table 3).

**Table 3:** Clinical characteristics of patients, the relationship of biomarkers and blood parameters Note: **: Correlation is significant at the 0.01 level; *: Correlation is significant at the 0.05 level; ***: Spearman’s Correlation; ALT: Alanine Aminotransferase; AST: Aspartate Aminotransferase; AUDIT: Alcohol Use Disorders Identification Test; BMI: Body Mass Index; CALLY: C-reactive protein-albumin-lymphocyte; CRP: C-Reactive Protein; GGT: Gamma Glutamyl Transferase; HDL: high-density lipoprotein; AIP: atherogenic index of plasma; LDL: low-density lipoprotein; SII: Systemic Immune Inflammation Index; SIRI: Systemic Inflammatory Response Index; TG: Triglyceride.

		Amount of alcohol use	Duration of alcohol use	AUDIT	SII^***^	SIRI^***^	AIP	CALLY
Age	r	0.471^**^	0.589^**^	0.371^**^	0.174	0.094	0.482^**^	-0.255
	p	0.000	0.000	0.006	0.208	0.501	0.000	0.063
BMI	r	0.039	-0.008	0.097	-0.282^*^	-0.360^**^	0.037	0.106
	p	0.780	0.952	0.486	0.038	0.007	0.793	0.447
Neutrophil	r	0.462^**^	0.483^**^	0.349^**^	0.561^**^	0.641^**^	0.273^*^	-0.226
	p	0.000	0.000	0.010	0.000	0.000	0.045	0.101
Lymphocyte	r	-0.060	0.108	-0.107	-0.426^**^	-0.123	0.028	0.219
	p	0.664	0.437	0.443	0.001	0.374	0.839	0.112
Monocyte^***^	r	0.056	-0.289^*^	0.126	-0.143	0.379^**^	0.108	-0.205
	p	0.686	0.034	0.363	0.302	0.005	0.439	0.138
Platelet	r	0.201	0.196	0.122	0.533^**^	0.218	0.191	-0.215
	p	0.145	0.156	0.379	0.000	0.114	0.167	0.118
Ferritin^***^	r	0.140	0.026	0.129	0.010	0.104	0.179	-0.104
	p	0.312	0.854	0.354	0.944	0.456	0.195	0.453
ALT^***^	r	0.141	0.058	0.072	-0.192	0.077	0.019	0.089
	p	0.308	0.679	0.605	0.164	0.581	0.894	0.524
AST ^***^	r	0.234	0.149	0.164	-0.018	0.204	0.093	-0.117
	p	0.088	0.282	0.236	0.896	0.139	0.503	0.398
GGT^***^	r	0.547^**^	0.303^*^	0.352^**^	0.122	0.315^*^	0.492^**^	-0.274^*^
	p	0.000	0.026	0.009	0.378	0.020	0.000	0.045
TG^***^	r	0.696^**^	0.364^**^	0.566^**^	0.334^*^	0.442^**^	0.809^**^	-0.499^**^
	p	0.000	0.007	0.000	0.014	0.001	0.000	0.000
HDL Cholesterol	r	-0.335^*^	-0.085	-0.308^*^	0.146	-0.112	-0.734^**^	0.275^*^
	p	0.013	0.543	0.023	0.291	0.419	0.000	0.044
LDL Cholesterol	r	-0.161	0.038	-0.121	-0.189	-0.107	0.097	0.240
	p	0.245	0.787	0.383	0.171	0.441	0.486	0.080
Total Cholesterol	r	0.099	0.309^*^	0.087	0.173	0.263	0.144	-0.055
	p	0.477	0.023	0.533	0.210	0.055	0.298	0.695
Albumin	r	-0.600^**^	-0.302^*^	-0.470^**^	-0.339^*^	-0.174	-0.387^**^	0.517^**^
	p	0.000	0.026	0.000	0.012	.0209	0.004	0.000
CRP^***^	r	0.675^**^	0.308^*^	0.625^**^	0.242	0.523^**^	0.589^**^	-0.965^**^
	p	0.000	0.023	0.000	0.078	0.000	0.000	0.000
amount of alcohol use	r	1	0.450^**^	0.749^**^	0.561^**^	0.509^**^	0.683^**^	-0.577^**^
	p		0.001	0.000	0.000	0.000	0.000	0.000
duration of alcohol use	r	0.450^**^	1	0.340^*^	0.345^*^	0.203	0.252	-0.160
	p	0.001		0.012	0.011	0.142	0.065	0.247
AUDIT	r	0.749^**^	0.340^*^	1	0.403^**^	0.437^**^	0.596^**^	-0.569^**^
	p	0.000	0.012		0.003	0.001	0.000	0.000
SII^***^	r	0.296^*^	0.288^*^	0.264	1	0.711^**^	0.258	-0.342^*^
	p	0.030	0.035	0.054		0.000	0.059	0.011
SIRI^***^	r	0.411^**^	0.109	0.379^**^	0.711^**^	1	0.423^**^	-0.533^**^
	p	0.002	0.431	0.005	0.000		0.001	0.000
AIP	r	0.683^**^	0.252	0.596^**^	0.258	0.423^**^	1	-0.442^**^
	p	0.000	0.065	0.000	0.059	0.001		0.001
CALLY index	r	-0.577^**^	-0.160	-0.569^**^	-0.342^*^	-0.533^**^	-0.442^**^	1
	p	0.000	0.247	0.000	0.011	0.000	0.001	

**Figure 1:**
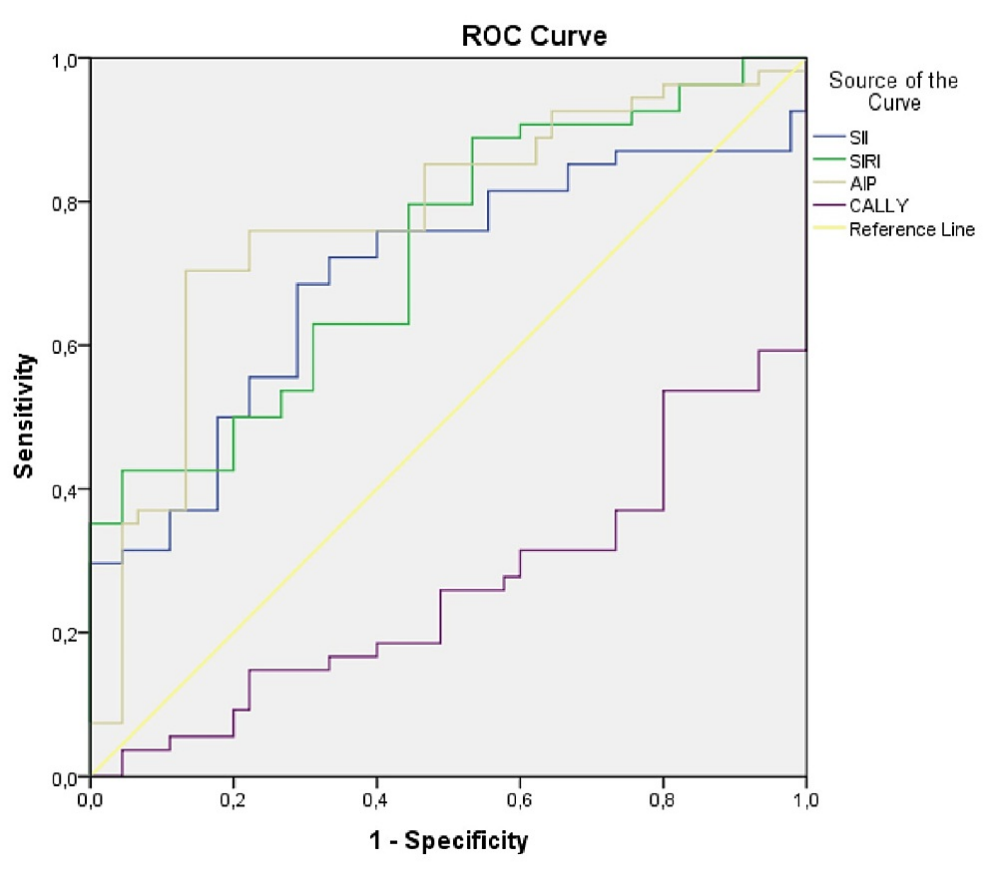
ROC curve analysis for SII, SIRI, AIP and CALLY index AIP: Atherogenic index of plasma (cut-off value 0.21, sensitivity %75.9, specificity %77.8); CALLY: C-reactive protein-albumin-lymphocyte (cut-off value 5.14, sensitivity %68.5, specificity %73.3); SII: Systemic immune inflammation index (cut-off value 384.48 sensitivity %68.5, specificity %71.1); SIRI: Systemic inflammatory response index (cut-off value 1.03, sensitivity %63, specificity %68.9).

ROC analysis was performed to reveal the role of SII, SIRI, AIP, and CALLY indices in distinguishing AUD and to determine the cut-off values. The analysis results showed that SII (AUC±SE; 95% CI; 0.695±0.054; 0.590-0.801), SIRI (AUC±SE; 95% CI; 0.730±0.050; 0.633-0.828), AIP (AUC±SE; 95% CI; 0.775±0.048; 0.680-0.870), and CALLY index (AUC±SE; 95% CI; 0.740±0.049; 0.643-0.836) could be used to identify AUD. It was shown that high values of SII, SIRI, and AIP, and low values of the CALLY index increased the likelihood of identifying AUD (Table 4, Figure 1).

**Table 4:** Area under the curve Note: a. Under the nonparametric assumption; b. Null hypothesis: true area = 0.5; p<0.05; Statistical significance level; CALLY: C-reactive protein-albumin-lymphocyte; AIP: Atherogenic index of plasma; SII: Systemic immune inflammation index; SIRI: Systemic inflammatory response index.

Test Result Variable(s)		Area	Std. Error^a^	Asymptotic Sig.^b^	Asymptotic 95% Confidence Interval	
					Lower Bound	Upper Bound
	SII	0.695	0.054	0.001	0.590	0.801
	SIRI	0.730	0.050	0.000	0.633	0.828
	AIP	0.775	0.048	0.000	0.680	0.870
	CALLY index	0.740	0.049	0.000	0.643	0.836

A linear regression analysis was conducted to investigate the effects of common variables in a mixed model including age, BMI, amount of alcohol consumed (standard drinks/per week), duration of alcohol use (moon), and total AUDIT score. According to the analysis results, in the model created for SII (B±SE; 17.28±6.31, p=0.009) and SIRI (B±SE; 0.038±0.017, p=0.026), only the amount of alcohol consumed was effective. In the model created for AIP, the amount of alcohol consumed (B±SE; 0.016±0.005, p=0.003) and age (B±SE; 0.008±0.003, p=0.013) were effective. In the model created for the CALLY index, the amount of alcohol consumed (B±SE; -0.181±0.090, p=0.049) and the total AUDIT score (B±SE; -0.152±0.075, p=0.049) were effective (Table 5).

**Table 5:** Linear regression analysis for SII, SIRI, AIP and CALLY index Note: p<0.05: Statistical significance level; AIP: Atherogenic index of plasma; AUDIT: Alcohol use disorders identification test; B: Regression coefficients; BMI: Body mass index; CALLY: C-Reactive protein-albumin-lymphocyte; CI: Confidence intervals; OR: Odds ratio; SII: Systemic immune inflammation index; SIRI: Systemic inflammatory response index.

Parameters	Independent variables	B (95%Cl)	OR	p value	R^2^/adjusted R^2^	p value for F change
SII	Model				0.353/0.286	0.001
	Age	1.162(-6.657/8.987)	0.047	0.766		
	BMI	-14.082(-34.043/5.878)	-0.175	0.162		
	amount of alcohol use	17.280(4.589/29.971)	0.517	0.009		
	duration of alcohol use	0.244(-0.591/1.078)	0.088	0.560		
	AUDIT	-0.451(-11.055/10.153)	-0.015	0.932		
SIRI	Model				0.316/0.245	0.002
	Age	-0.008(-0.028/0.013)	-0.120	0.466		
	BMI	-0.036(-0.088/0.017)	-0.174	0.178		
	amount of alcohol use	0.038(0.005/0.072)	0.445	0.026		
	duration of alcohol use	0.000(-0.002/0.002)	0.017	0.912		
	AUDIT	0.012(-0.016/0.040)	0.160	0.383		
AIP	Model				0.550/0.503	0.000
	Age	0.008(0.002/0.014)	0.344	0.013		
	BMI	-0.007(-0.023/0.009)	-0.093	0.374		
	amount of alcohol use	0.016(0.005/0.026)	0.487	0.003		
	duration of alcohol use	-0.001(-0.001/0.000)	-0.235	0.068		
	AUDIT	0.006(-0.003/0.014)	0.192	0.197		
CALLY index	Model				0.420/0.359	0.000
	Age	-0.044(-0.155/0.068)	-0.118	0.434		
	BMI	0.223(-0.061/0.508)	0.185	0.121		
	amount of alcohol use	-0.181(-0.362/-0.001)	-0.361	0.049		
	duration of alcohol use	0.008(-0.004/0.020)	0.188	0.195		
	AUDIT	-0.152(-0.303/-0.001)	-0.337	0.049		

## Discussion

This study evaluated the relationship between AUD and SII, SIRI, AIP, and CALLY indices, which are considered as inflammatory, immunonutritive, and cardiovascular markers based on the combined evaluation of blood cell count and biochemical parameters. Our results showed that compared to controls, those with AUD had increased AST and GGT activities, decreased albumin levels, and systemic inflammation occurred. It was shown that AUD has different systemic inflammation indicators such as SII, SIRI. The amount of alcohol consumed was a predictor for high SII and SIRI levels. The laboratory results of those with AUD were consistent with atherogenic dyslipidemia compared to controls; significantly higher TG and total cholesterol levels and AIP value. Also, the CALLY index, which evaluates immune function, inflammation, and nutritional status together, was found to be significantly lower in patients compared to controls. The amount of alcohol consumed and the AUDIT score were predictors for the low CALLY index. These results support studies in the literature reporting the adverse effect of heavy alcohol consumption on liver functions, increase in inflammatory response, and serum lipid profile [4,13].

The majority of patients were smokers, and some had a history of illicit substance use. It has been reported that alcohol is the substance that most often leads to addiction among the elderly and that the male gender is a risk factor for alcohol use [24]. In our recent study, it was revealed that smoking is common among alcohol users, some alcohol users have a history of substance use, and the BMI of alcohol users is lower compared to non-alcohol users [25]. It has been reported that low to moderate alcohol consumers tend to increase fat mass, chronic alcohol consumers tend to decrease fat mass, the waist/hip ratio increases with chronic alcohol consumption, and heavy chronic alcohol consumption leads to pathological lipoatrophy or redistribution of fat through changes in lipolytic and lipogenic balance [26]. The literature also includes different effects of different types of alcohol on BMI (wine; weight gain in women, negative relationship with BMI in men) [26].

In those with AUD, AST, GGT activities, ferritin, TG, and total cholesterol levels were significantly higher. These results were consistent with the literature [18,27,28]. It has been reported that ferritin levels vary in response to the stage of liver disease [18]. Long-term high-level alcohol consumption is known to cause mild increases in liver enzyme activities, as well as more severe liver diseases such as alcoholic fatty liver, cirrhosis, hepatitis, or alcoholic hepatic encephalopathy [7]. Abnormalities in liver enzyme activities (GGT, AST, or ALT) are often the first clinical signs of excessive alcohol consumption [18]. AST and ALT are direct markers of liver damage and inflammation [3]. GGT activity is a useful biomarker for detecting relapse as well as heavy alcohol consumption in those with AUD [3]. It has been reported that high GGT activity in men is associated with increased cardiovascular risk, and a significant correlation has been found between LDL and GGT levels [18]. We found a significant relationship between GGT activity and LDL (r=0.320, p=0.018) and AIP (r=0.492, p<0.001) values. This result is interesting because there is a positive correlation between high GGT activity and chronic alcohol consumption (18). This could explain the findings related to LDL cholesterol values, i.e., the atherogenic dyslipidemia we demonstrated in the study.

Many studies have shown that alcohol is effective on the immune system and inflammation. Alcohol use has been associated with hematological markers based on CRP and white blood cell count and subtypes (neutrophils, lymphocytes, monocytes, and platelets) ratios (SII, SIRI) [2,13,16]. In our study, neutrophil and CRP levels, SII, and SIRI were significantly higher in those with AUD. It is increasingly accepted that systemic inflammation initiates and exacerbates the pathological process of chronic diseases. The immune system and inflammatory processes play a key role in the pathogenesis of atherosclerosis, as shown in the literature [10]. In our study, the laboratory results of those with AUD were consistent with atherogenic dyslipidemia; TG and total cholesterol levels and AIP values were significantly higher in patients. Our results were consistent with the results of a recent study evaluating atherosclerotic risk in which 45 male patients with AUD were included [4]. We found significant positive correlations between patients’ AIP value and inflammatory markers such as neutrophil, CRP, and SIRI. The amount of alcohol consumed (standard drinks/per week) was a predictor for high SII, SIRI, and AIP levels. It has been reported that monocytes and neutrophils contribute to abnormal coronary plaque formation, induce atherosclerotic plaque rupture and thrombosis, activate the inflammatory response, and increase CVD risk [10,29]. In a 20-year follow-up study of 42,875 adults, it was found that high SII or SIRI was closely associated with increased cardiovascular mortality and all-cause mortality in the general population [10].

In our study, we found albumin levels to be significantly low, while lymphocyte levels were lower in patients, although not to a significant degree. A decrease in the number of lymphocytes leads to a decrease in the functions of the immune system and causes immunological dysfunction [10]. It is also stated in the literature that lymphocytes prevent the progression of atherosclerosis, and lymphopenia positively correlates with the frequency of heart failure and poor prognosis in patients with acute coronary syndrome [29]. It has been reported that serum albumin levels change in response to the stage of liver disease, and synthesis rates decrease in those with liver disease [18]. The CALLY index is a biomarker developed to reflect the level of inflammation, nutritional status, and immune function. It has been defined as a prognostic biomarker in cancer patients by Iida and colleagues [17]. In our study, the CALLY index was significantly lower in those with AUD. In our study, low values of the CALLY index were associated with higher GGT activity and TG levels, increased inflammatory markers (SII-SIRI) and AIP, greater alcohol consumption amount, and total AUDIT score. The amount of alcohol consumed and the total AUDIT score were predictors of a lower CALLY index. We saw in the literature that there are studies examining the relationship of the CALLY index with tumors [17,30], but we did not find any study investigating its relationship with AUD or even psychiatric disorders. Since the CALLY index is associated with factors related to AUD as well as markers based on inflammation, it is likely a biomarker as good as or better than SII, SIRI, and AIP. In AUD, more comprehensive studies to be conducted in the future with the SII, SIRI, AIP, and CALLY index can provide useful inflammation-based information for the optimization of the severity of AUD, complications, monitoring of patients’ treatment response, and planning of therapeutic strategy.

This study has both strengths and limitations. It is a retrospective study and some of the data was based on personal reporting, so potential bias could not be completely excluded. The cross-sectional design of the study prevents the establishment of causal relationships between biomarkers and outcomes related to addiction. Another limitation of our study could be that participants were not subjected to radiological evaluation for atherosclerotic findings, despite having no known cardiovascular diseases. Furthermore, both the prevalence of AUD and the risk of cardiovascular disease are higher in men than in women - and the study only included male cases - which is another limitation of our study. The fact that the number of patients who smoke is higher than the controls is another limitation of our study. Not examining the relationship of different types of alcohol (such as beer, wine, rum) with markers, our small sample size, and the fact that some of our patients have used illegal substances in the past are other limitations of our study. Although addiction is a comorbid disease by nature, the exclusion of patients with major comorbidities is both a limitation and a strength of our study. On the other hand, our results revealed strong relationships between inflammatory, immunonutritive, and atherogenic dyslipidemia indicators such as SII, SIRI, AIP, and CALLY index levels and AUD.

## Conclusions

Our results revealed that, compared to the controls, those with AUD had increased AST and GGT activities, neutrophil, ferritin, and CRP levels. It was shown that AUD has different systemic inflammation indicators such as SII and SIRI. The amount of alcohol consumed was a predictor for high SII and SIRI levels. The laboratory results of those with AUD were consistent with atherogenic dyslipidemia; significantly higher TG and total cholesterol levels and AIP value were detected compared to the controls. Also, the CALLY index, which evaluates immune function, inflammation, and nutritional status together, was significantly lower in those with AUD. The amount of alcohol use and the total AUDIT score were predictors for a low CALLY index. The results of this study support that AUD is a chronic inflammatory psychiatric disorder. New inflammatory, immunonutritive, and cardiovascular biomarkers; SII, SIRI, AIP, and CALLY index may be promising clinical tools to evaluate the severity, possible complications, and treatment response of AUD, therefore, we suggest conducting longitudinal and well-designed studies on larger patient groups. In this study, which we aimed to reflect a real community sample, it should not be forgotten that the study results may not represent the entire community.

## References

[1] Köroğlu E (2013). Madde ile İlişkili Bozukluklar ve Bağımlılık Bozuklukları. In Ruhsal Bozuklukların Tanısal ve Sayımsal Elkitabı.

[2] Adams C, Conigrave JH, Lewohl J, Haber P, Morley KC (2020). Alcohol use disorder and circulating cytokines: a systematic review and meta-analysis. Brain Behav Immun.

[3] Harris JC, Leggio L, Farokhnia M (2021). Blood biomarkers of alcohol use: a scoping review. Curr Addict Rep.

[4] Senat A, Kabadayi-Sahin E, Sogut I, Duymaz T, Erel O (2023). Evaluation of atherosclerotic risk by oxidative contributors in alcohol use disorder. Clin Psychopharmacol Neurosci.

[5] Huang S, Li J, Shearer GC (2017). Longitudinal study of alcohol consumption and HDL concentrations: a community-based study. Am J Clin Nutr.

[6] Piano MR (2017). Alcohol's effects on the cardiovascular system. Alcohol Res.

[7] Morcuende A, Navarrete F, Nieto E, Manzanares J, Femenía T (2021). Inflammatory biomarkers in addictive disorders. Biomolecules.

[8] Ross R (1999). Atherosclerosis--an inflammatory disease. N Engl J Med.

[9] Ridker PM (2001). High-sensitivity C-reactive protein: potential adjunct for global risk assessment in the primary prevention of cardiovascular disease. Circulation.

[10] Xia Y, Xia C, Wu L, Li Z, Li H, Zhang J (2023). Systemic immune inflammation index (SII), system inflammation response index (SIRI) and risk of all-cause mortality and cardiovascular mortality: a 20-year follow-up cohort study of 42,875 US adults. J Clin Med.

[11] Debnath M, Doyle K, Langan C, McDonald C, Leonard B, Cannon D (2011). Recent advances in psychoneuroimmunology: inflammation in psychiatric disorders. Translational Neuroscience.

[12] Niemelä O, Halkola AS, Bloigu A, Bloigu R, Nivukoski U, Pohjasniemi H, Kultti J (2022). Blood cell responses following heavy alcohol consumption coincide with changes in acute phase reactants of inflammation, indices of hemolysis and immune responses to ethanol metabolites. Int J Mol Sci.

[13] Kok Kendirlioglu B, Arat Celik HE, Buyuksandalyaci Tunc AE, Ozmen M, Corekli Kaymakcı E, Demir S, Kuçukgoncu S (2024). Lymphocyte-related ratios, systemic immune-inflammatory and systemic inflammatory response index in alcohol use disorder. J Immunoassay Immunochem.

[14] Orum MH, Kara MZ (2020). Platelet to lymphocyte ratio (PLR) in alcohol use disorder. J Immunoassay Immunochem.

[15] Ruwel AG, Scherer JN, Silvello D, Kessler FH, von Diemen L, Schuch JB (2024). Hematological inflammatory biomarkers in patients with alcohol and cocaine use disorders. Trends Psychiatry Psychother.

[16] Büyükbaş S, İnal A (2007). Erkeklerde Aşırı Alkol Kullanımının C Reaktif Protein ve Alfa-1 Antitripsin Üzerine Olan Etkileri. Van Tıp Dergisi.

[17] Iida H, Tani M, Komeda K (2022). Superiority of CRP-albumin-lymphocyte index (CALLY index) as a non-invasive prognostic biomarker after hepatectomy for hepatocellular carcinoma. HPB (Oxford).

[18] Niemelä O (2016). Biomarker-based approaches for assessing alcohol use disorders. Int J Environ Res Public Health.

[19] World Health Organization (2024). Brief intervention for hazardous and harmful drinking: a manual for use in primary care. https://www.who.int/publications/i/item/brief-intervention-for-hazardous-and-harmful-drinking-(audit).

[20] Babor TF, Higgins-Biddle JC, Saunders JB, Monteiro M, World Health Organization (2024). AUDIT : the alcohol use disorders identification test: guidelines for use in primary health care. https://www.who.int/publications/i/item/WHO-MSD-MSB-01.6a.

[21] Saatçioğlu Ö, Evren C, Çakmak D (2002). Alkol kullanım bozuklukları tanıma testinin geçerliği ve güvenirliği. Türkiye’de Psikiyatri.

[22] (2013). Conversion factors of Conventional units to SI units. Kidney International Supplements.

[23] George D, Mallery M (2010). SPSS for Windows Step by Step: A Simple Guide and Reference (17.0 update, 10a ed.). SPSS for Windows Step by Step: A Simple Guide and Reference, 17.0 update (10a.

[24] Çavuşoğlu Ç, Demirkol ME (2018). Yaşlılarda Bağımlılık. Bağımlılık Dergisi.

[25] Gurbuzer N, Akkus S (2024). Tobacco, alcohol, illegal drug and substance use, sociodemographic variables and psychological distress symptoms in individuals aged 50-65. Annals of Medical Research.

[26] Steiner JL, Lang CH (2017). Alcohol, adipose tissue and lipid dysregulation. Biomolecules.

[27] Chen CH, Walker J, Momenan R, Rawlings R, Heilig M, Hommer DW (2012). Relationship between liver function and brain shrinkage in patients with alcohol dependence. Alcohol Clin Exp Res.

[28] Coulbault L, Laniepce A, Segobin S, Boudehent C, Cabé N, Pitel AL (2022). Trimethylamine N-oxide (TMAO) and indoxyl sulfate concentrations in patients with alcohol use disorder. Nutrients.

[29] Dziedzic EA, Gąsior JS, Tuzimek A, Paleczny J, Junka A, Dąbrowski M, Jankowski P (2022). Investigation of the associations of novel inflammatory biomarkers-systemic inflammatory index (SII) and systemic inflammatory response index (SIRI)-with the severity of coronary artery disease and acute coronary syndrome occurrence. Int J Mol Sci.

[30] Yang M, Lin SQ, Liu XY (2023). Association between C-reactive protein-albumin-lymphocyte (CALLY) index and overall survival in patients with colorectal cancer: from the investigation on nutrition status and clinical outcome of common cancers study. Front Immunol.

